# The Envelope Gene of Transmitted HIV-1 Resists a Late Interferon Gamma-Induced Block

**DOI:** 10.1128/JVI.02254-16

**Published:** 2017-03-13

**Authors:** Suzannah J. Rihn, Toshana L. Foster, Idoia Busnadiego, Muhamad Afiq Aziz, Joseph Hughes, Stuart J. D. Neil, Sam J. Wilson

**Affiliations:** a MRC—University of Glasgow Centre for Virus Research, Institute of Infection, Inflammation and Immunity, University of Glasgow, Glasgow, United Kingdom; b Department of Infectious Diseases, King's College London Faculty of Life Sciences and Medicine, Guy's Hospital, London, United Kingdom; Ulm University Medical Center

**Keywords:** envelope, HIV-1, interferons, restriction factors, transmitted/founder, type II interferon

## Abstract

Type I interferon (IFN) signaling engenders an antiviral state that likely plays an important role in constraining HIV-1 transmission and contributes to defining subsequent AIDS pathogenesis. Type II IFN (IFN-γ) also induces an antiviral state but is often primarily considered to be an immunomodulatory cytokine. We report that IFN-γ stimulation can induce an antiviral state that can be both distinct from that of type I interferon and can potently inhibit HIV-1 in primary CD4^+^ T cells and a number of human cell lines. Strikingly, we find that transmitted/founder (TF) HIV-1 viruses can resist a late block that is induced by type II IFN, and the use of chimeric IFN-γ-sensitive/resistant viruses indicates that interferon resistance maps to the *env* gene. Simultaneously, *in vitro* evolution also revealed that just a single amino acid substitution in the envelope can confer substantial resistance to IFN-mediated inhibition. Thus, the *env* gene of transmitted HIV-1 confers resistance to a late block that is phenotypically distinct from blocks previously described to be resisted by *env* and is therefore mediated by unknown IFN-γ-stimulated factor(s) in human CD4^+^ T cells and cell lines. This important unidentified block could play a key role in constraining HIV-1 transmission.

**IMPORTANCE** The human immune system can hinder invading pathogens through interferon (IFN) signaling. One consequence of this signaling is that cells enter an antiviral state, increasing the levels of hundreds of defenses that can inhibit the replication and spread of viruses. The majority of HIV-1 infections result from a single virus particle (the transmitted/founder) that makes it past these defenses and colonizes the host. Thus, the founder virus is hypothesized to be a relatively interferon-resistant entity. Here, we show that certain HIV-1 envelope genes have the unanticipated ability to resist specific human defenses mediated by different types of interferons. Strikingly, the envelope gene from a founder HIV-1 virus is far better at evading these defenses than the corresponding gene from a common HIV-1 lab strain. Thus, these defenses could play a role in constraining the transmission of HIV-1 and may select for transmitted viruses that are resistant to this IFN-mediated inhibition.

## INTRODUCTION

Humans have evolved to possess a diverse arsenal of antiretroviral defenses. Despite this, the human immune system is unable to eradicate an established human immunodeficiency virus (HIV) infection. Following infection, individuals mount vigorous immune responses targeting HIV-1 (1). While these responses are unable to clear systemic HIV-1 infection, the timing and magnitude of the innate and acquired immune responses still play a key role in shaping disease progression and pathogenesis.

A major component of innate immunity is the interferon (IFN) system, which is comprised of a diverse family of related cytokines that for humans includes 17 type I IFNs (13 IFN-α subtypes, as well as IFN-β,- κ, -ε, and -ω), one type II IFN (IFN-γ), and 4 type III IFNs (IFN-λ1 to IFN-λ4). Research into how IFNs inhibit retroviruses has largely focused on type I IFNs as type I/III IFNs are often perceived as the major antiviral IFNs, whereas IFN-γ is frequently considered solely as an immunomodulatory player (2). To this end, it is now well established that type I IFNs inhibit infection and replication of HIV-1 and related primate lentiviruses both *in vitro* (3–12) and *in vivo* (13) (recently reviewed by Doyle et al. [2]). Notably, HIV-1-infected individuals treated with IFN-α experience significant, albeit transient, reductions in viral loads (13). Similarly, rhesus macaques administered IFN-α can resist simian immunodeficiency virus (SIV) infection (14). In addition, transmitted HIV-1 is proposed to be relatively IFN resistant (15, 16) (although this is not universally observed [17]). Despite this, IFNs are not always beneficial to the host, and repeated IFN administration in primate models (14), or persistent stimulation in chronically infected patients, is associated with poorer clinical outcome (18, 19). Thus, although IFN responses do not eradicate systemic HIV-1, there is great interest in understanding how IFNs might shape susceptibility to HIV-1 infection and subsequent progression to AIDS.

Over the last decade, much of the attention paid to the ability of type I IFNs to inhibit HIV-1 has focused on restriction factors, including TRIM5/TRIMCyp (20, 21), APOBEC3s (22), tetherin/BST2 (23), and SAMHD1 (24, 25). These interferon-stimulated genes (ISGs) represent powerful barriers that primate lentiviruses must evade or overcome in order to thrive within human populations (26), and even successful viruses do not always completely escape inhibition by these factors (27). Alongside the restriction factors, a growing number of other ISGs have been identified as being capable of inhibiting HIV-1 but are not fully evaded or antagonized in natural settings. These “resistance factors” include IFITMs (28–30), GBP5 (31), and Mx2/MxB (32, 33). Importantly, these known resistance factors, along with the established restriction factors, still cannot fully explain the IFN-mediated inhibition of HIV-1 observed *in vitro* (2). Thus, there is great interest in understanding the molecular details of how IFNs might constrain HIV transmission, acute viral replication, pathogenesis, or even the pandemic potential of geographically restricted HIVs (13–16, 30, 34, 35).

Despite this predominant focus on type I IFNs and type I ISGs, reports in the last century demonstrated that IFN-γ treatment can also confer substantial antiretroviral activity *in vitro* (5, 9, 36, 37). Recently, this concept has been revisited with the observations that some antiretroviral ISGs, such as GBP5 and IDO1, are most strongly upregulated by IFN-γ (31, 38). Although the antiretroviral potential of IFN-γ has been reported, and patients mount robust IFN-γ responses following HIV-1 infection (1), the clinical significance of these observations is currently unclear.

Here we show that IFN-γ has anti-HIV-1 activity in primary CD4^+^ T cells and a number of common cell lines and can induce strong early and late blocks to HIV-1 replication. IFN-γ can induce a divergent antiviral state from type I IFNs, and potent IFN-γ-induced early block(s) to HIV-1 infection can be entirely independent of Mx2. Surprisingly, not all HIVs are equally susceptible to IFN-γ-mediated inhibition, and certain HIV-1 and HIV-2 strains can resist inhibition. Crucially, HIV-1 transmitted/founder (TF) viruses are strikingly resistant to a late IFN-γ-stimulated block. Using two independent approaches, we map IFN resistance to the HIV-1 *env* gene. Notably, a single amino acid substitution in the envelope protein conferred substantial resistance to IFN-mediated inhibition. Thus, the *env* gene from transmitted HIV-1 can resist unidentified IFN-induced factor(s), and this ability has been lost in multiple common lab strains. The potent inhibition observed here suggests that type II IFN may play an underappreciated role in limiting HIV-1 transmission and disease progression in a fashion similar to that of type I IFNs.

## RESULTS

### IFN-γ and IFN-α induce divergent antiviral states in THP-1 cells.

**FIG 1:**
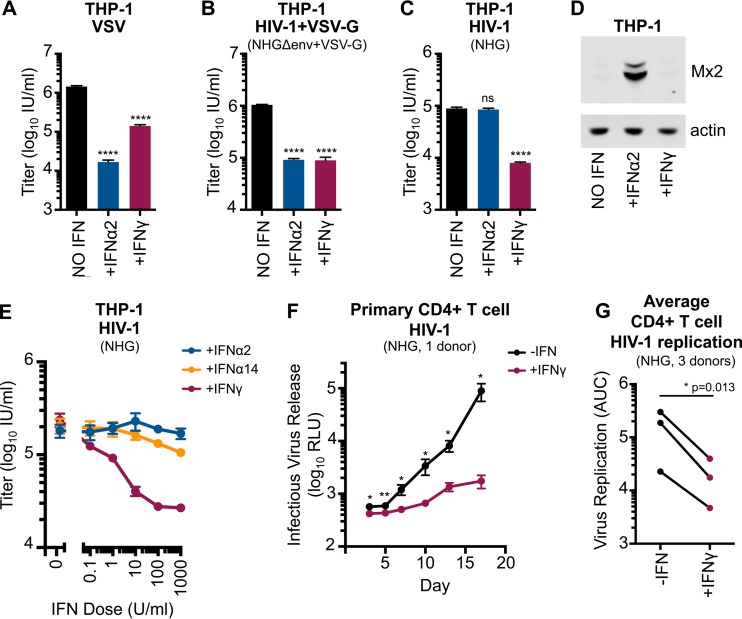
IFN-γ can induce a divergent antiretroviral state and can inhibit HIV-1 in primary CD4^+^ T cells. (A to C) THP-1 cells were treated or untreated with 1,000 U/ml of IFN-α2 or IFN-γ for 24 h prior to titrated challenge with VSV (VSV-ΔG-GFP.VSV-G), VSV-G pseudotyped, single-cycle, HIV-1 (NHGΔenv-GFP+VSV-G), or HIV-1 (NHG). At 18 h postinfection cells infected with NHG were treated with dextran sulfate to limit infection to a single cycle. At 48 h postinfection, all cells were fixed, and the infectious titers were determined using flow cytometry. (D) Mx2 and actin expression in lysates from IFN-α2- or IFN-γ-treated cells were assessed using Western blotting (WB). (E) IFN dose-response determinations for IFN-α2, IFN-α14, and IFN-γ were performed as described for panel C, including addition of dextran sulfate at 18 h postinfection. (F) The infectious HIV-1 (NHG) yield from human primary CD4^+^ T cells with or without IFN-γ (1 donor, *n* = 4), at an MOI of 0.05, at 3 to 17 days (determined by TZM-bl assay). (G) Primary CD4^+^ T cell replication with or without IFN-γ for three donors for 3 to 13 days at an MOI of 0.05 to 0.1. Shown are area under the curve (AUC) values from individual infectious yield growth curves (typical curves are shown in panel F). Errors bars indicate SEM. Statistical analyses were performed using unpaired (A to C and F) or paired (G) two-tailed *t* tests (****, *P* < 0.0001; **, *P* < 0.01; *, *P* < 0.05; ns, not significant, *P* > 0.05; *n* = 3 to 5). RLU, relative light units.

Our recent demonstration of the anti-HIV-1 activity of IDO1 (38) led us to further consider the IFN-γ-induced antiviral state. We initially examined THP-1 cells, which exhibit a strong IFN-induced block to HIV-1 infection (32). To confirm the induction of an antiviral state, THP-1 cells were pretreated with IFN-α2 or IFN-γ for 24 h before challenge with a single-cycle vesicular stomatitis virus with a green fluorescent protein (VSV-GFP) reporter. IFN-α2 and IFN-γ both potently inhibited VSV infection although the inhibition displayed by IFN-α2 against VSV was greater (Fig. 1A). In parallel, we observed that IFN-α2 and IFN-γ conferred similar levels of protection (∼10-fold) from a single cycle of VSV G protein (VSV-G) pseudotyped HIV-1 infection (Fig. 1B). Finally, we considered the ability of a proviral clone of HIV-1 (NHG, a chimera of NL4-3 and HXB that encodes GFP in place of *nef*) to infect THP-1 cells stimulated with IFN-α2 or IFN-γ. Strikingly, while IFN-γ conferred ∼10-fold protection from HIV-1 infection in a single cycle, IFN-α2 did not induce protection (Fig. 1C). Thus, IFN-α2 induced more potent anti-VSV activity while IFN-γ conferred stronger anti-HIV-1 activity in THP-1 cells. Together, these data demonstrate that IFN-γ and IFN-α can induce phenotypically divergent antiviral states that inhibit distinct spectra of viruses.

Mx2 is known to mediate an early block to HIV-1 infection (32, 33). Mx2 expression was potently upregulated in THP-1 cells stimulated with IFN-α2 (Fig. 1D), whereas IFN-γ did not induce Mx2 expression. Importantly, the robust upregulation of Mx2 induced by IFN-α2 in THP-1 cells did not inhibit HIV-1 (Fig. 1C). Thus, IFN-γ can confer potent early protection against HIV-1 infection that is independent of Mx2.

Considerable variation in the potency of anti-HIV-1 activity conferred by type I IFNs has been reported (39). Thus, we also considered the ability of IFN-α14, which has potent anti-HIV-1 activity (39), alongside IFN-α2 and IFN-γ, to induce protection from HIV-1 infection in THP-1 cells. Notably, only IFN-γ induced substantial protection in a single cycle of HIV-1 infection (Fig. 1E) in THP-1 cells.

### IFN-γ-induced blocks in primary cells and human cell lines.

The divergent IFN-γ-induced antiviral state in THP-1 cells led us to examine whether IFN-γ also inhibited HIV-1 replication in primary cells. IFN-γ treatment potently inhibited HIV-1 replication in CD4^+^ T cells, suggesting that this inhibition may be relevant *in vivo* (Fig. 1F and G). Still, it is not clear whether this IFN-γ-mediated inhibition occurs directly or indirectly, as it is possible that a minority of contaminating cells (such as monocytes or dendritic cells) could respond to IFN-γ by secreting proinflammatory cytokines that induce an antiviral state in neighboring CD4^+^ T cells. Nevertheless, the strength of the inhibitory phenotype warranted further investigation. Since the mechanistic basis of IFN inhibition is typically dissected using common cell lines (32, 33) and these cultures are more uniform, we used a panel of human cell lines to examine the capacity of IFN-γ to either protect cells from HIV-1 infection or to inhibit the production of infectious progeny. We opted to treat our panel of cell lines with 1,000 units/ml of IFN-γ (a relatively high dose) to ensure that we did not overlook cell lines exhibiting inhibitory phenotypes.

**FIG 2:**
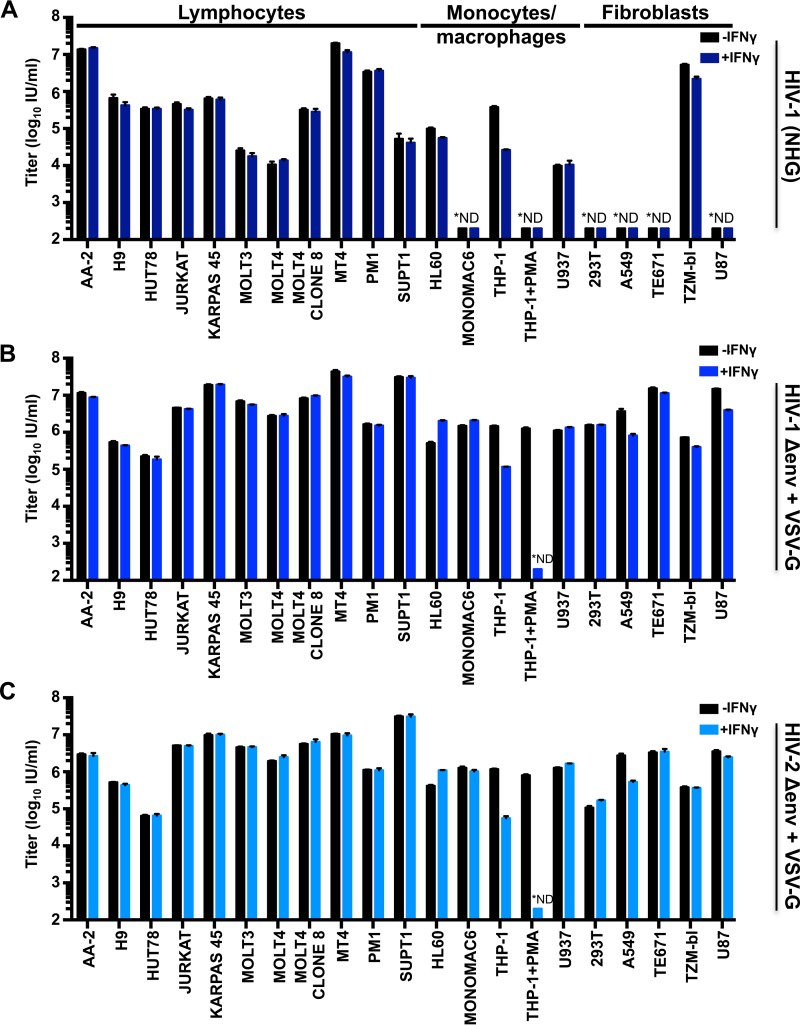
Early blocks to primate lentivirus infection in IFN-γ-treated cells are unusual in cultured human cells. Human cell lines were untreated or treated with 1,000 U/ml of IFN-γ for 24 h prior to titrated challenge with HIV-1 (NHG) (A), VSV-G pseudotyped HIV-1 (NHGΔenv-GFP) (B), or VSV-G pseudotyped HIV-2 (HIV-2_ROD_Δenv-GFP) (C). Cells infected with NHG were treated with dextran sulfate at 18 h postinfection to limit infection to a single cycle. At 48 h postinfection, all cells were fixed and analyzed as described in the legend of Fig. 1A to C. *ND, not detectable. Error bars indicate SEM (*n* = 3 to 5).

**FIG 3:**
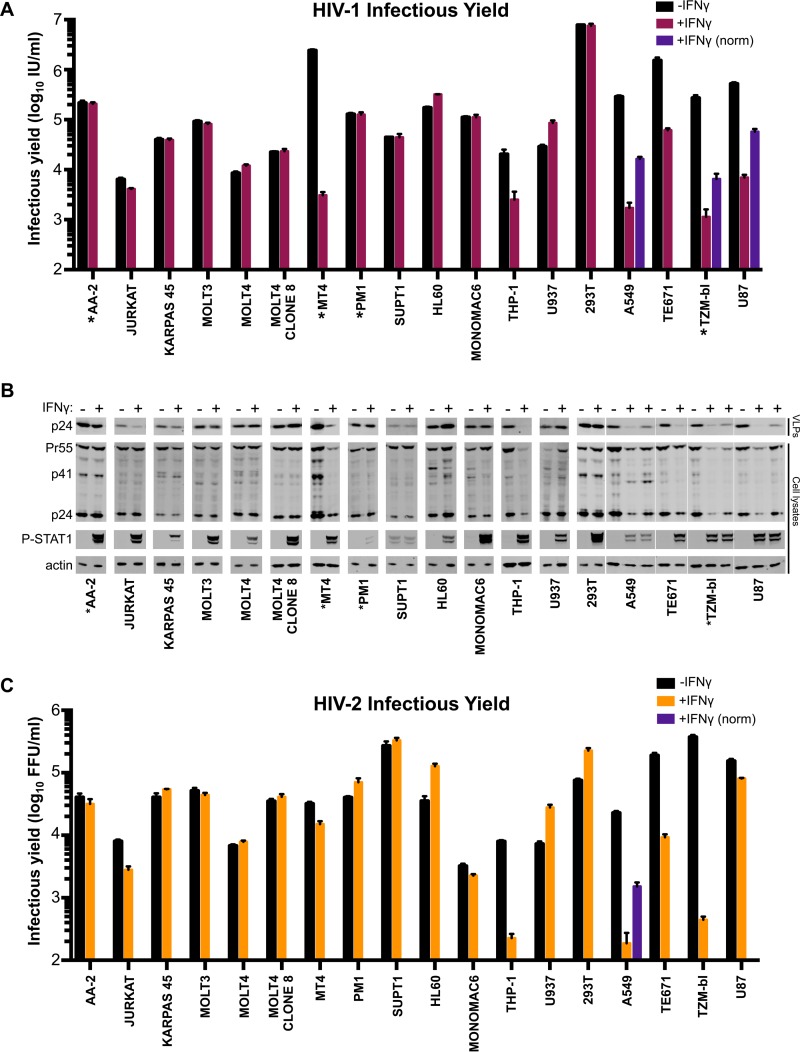
Multiple human cell lines exhibit late IFN-γ-induced blocks to HIV replication. (A and C) A panel of human cell lines was pretreated (or untreated) with 1,000 U/ml of IFN-γ for 24 h prior to challenge with NHG (denoted by * in A and B), VSV-G pseudotyped NHG (A and B), or VSV-G pseudotyped ROD10 (C). For cells in which the incoming block (Fig. 2B and C) was ≥2.5-fold [A549 (HIV-1 and HIV-2), TZM-bl (HIV-1), and U87 (HIV-1) cells], the weak incoming blocks were accounted for by increasing the inoculum in proportion to the strength of the incoming block [+IFN-γ (norm), although this was not achievable in THP-1 cells (excessive virus volume)]. The infectious yield was determined 46 to 48 h postinfection through titration of the harvested cell-free virus-containing supernatant onto either MT4 cells (A) or TZM-bl cells (C). (B) HIV-1 particulate capsid abundance (p24) in the supernatant (VLPs) as well as cellular Gag (Pr55), capsid (p24), phosphorylated STAT1, and host actin expression in the HIV-1 producer cells (A) were monitored by Western blotting. All error bars indicate SEM (*n* = 3 to 5).

As we were interested in factors that specifically target HIV-1, we compared the ability of IFN-γ treatment to inhibit HIV-1 and HIV-2 either early (Fig. 2) or late (Fig. 3) in the viral life cycle. Because not all of our cell lines could be efficiently infected with HIV-1 (Fig. 2A, NHG) and few were efficiently infected with HIV-2 (ROD10), we also utilized VSV-G pseudotyped reporter viruses (Fig. 2B and C). For the early (or incoming) inhibition highlighted in Fig. 2, all assays were limited to a single cycle through the use of either envelope-defective proviral clones or the addition of dextran sulfate following infection. Although modest protection from incoming infection (i.e., an early block) was observed in some cell lines (≤5-fold), predominantly fibroblasts, only IFN-γ-stimulated THP-1 cells were substantially protected from HIV-1 and HIV-2 infection (∼10-fold). Strikingly, phorbol myristate acetate (PMA) treatment of THP-1 cells greatly enhanced (>1,000-fold) this early block (Fig. 2), with the levels of infection being too low for us to accurately enumerate following IFN-γ treatment. Nevertheless, IFN-γ-induced protection from infection appears unusual in human cell lines and is equally effective against HIV-1 and HIV-2 (Fig. 2). In general, the rarity of IFN-γ-induced early blocks to HIV infection makes it difficult to assess whether HIV-2 might be more sensitive than HIV-1 to IFN-γ-stimulated defenses, as is the case for IFN-α (34).

Type I IFNs are known to induce both early (10) and late blocks to HIV-1 replication (6). Similarly, single ISGs can also confer either early (20) or late blocks to HIV-1 replication (40). Therefore, we also considered the ability of IFN-γ to inhibit later stages of the HIV-1 life cycle. To do this, virus-containing supernatants were harvested at 46 to 48 h postinfection as nascent virions from the first round of infection were abundant in the supernatant at this time, but progeny virions from subsequent rounds of infection had not yet accumulated (41). This allowed us to measure the yield of infectious HIV-1 or HIV-2 resulting from ∼1 round of infection (multiplicity of infection [MOI] of 0.5, determined using data from the experiment shown in Fig. 2).

Unlike most cell lines, a substantial IFN-γ-mediated reduction in infectious HIV-1 yield was observed in MT4, THP-1, A549, TE671/RD, TZM-bl, and U87 cells (Fig. 3A). HIV-1 production from IFN-γ-treated THP-1 cells was reduced by a magnitude (∼10-fold) almost identical to that of the early block to infection observed in these cells (Fig. 1C, 2A, and 3A). Thus, the diminished titer of HIV-1 produced by these cells is likely caused by the early/incoming block (Fig. 1C and E and 2A) rather than by a reduced capacity for HIV-1 replication (a late/outgoing/production block). Therefore, in THP-1 cells, IFN-γ induces inhibition early (but not late) in the HIV-1 life cycle. In contrast, IFN-γ-treated MT4, TE671, A549, TZM-bl, and U87 cells potently inhibited (∼10- to 800-fold) the production of infectious HIV-1 (Fig. 3A), even when weak early blocks in these cells were overcome by increasing the initial inoculum (in direct proportion to the incoming block) (Fig. 3A, purple bars). Although the IFN-γ receptor is expressed on nearly all cell types (reviewed in reference 42), signaling responses can vary enormously (43). Thus, we examined the levels of activated STAT1 (phosphorylated at residue 701) in our panel of cell lines. Nearly all cell lines responded robustly to IFN-γ treatment although KARPAS 45, PM1, and SUPT1 responded poorly, providing a likely explanation for the lack of IFN-γ-mediated inhibition of HIV-1 in these cells. Intriguingly, SUPT1 cells reproducibly exhibited STAT1 phosphorylation in the absence of cytokine treatment. More importantly, however, many of the cell lines that responded robustly to IFN-γ treatment did not inhibit HIV-1 replication. Thus, the factor(s) responsible for IFN-γ-stimulated HIV-1 inhibition in select cell lines are likely not induced or not competent to inhibit HIV-1 in multiple other cell lines.

**FIG 4:**
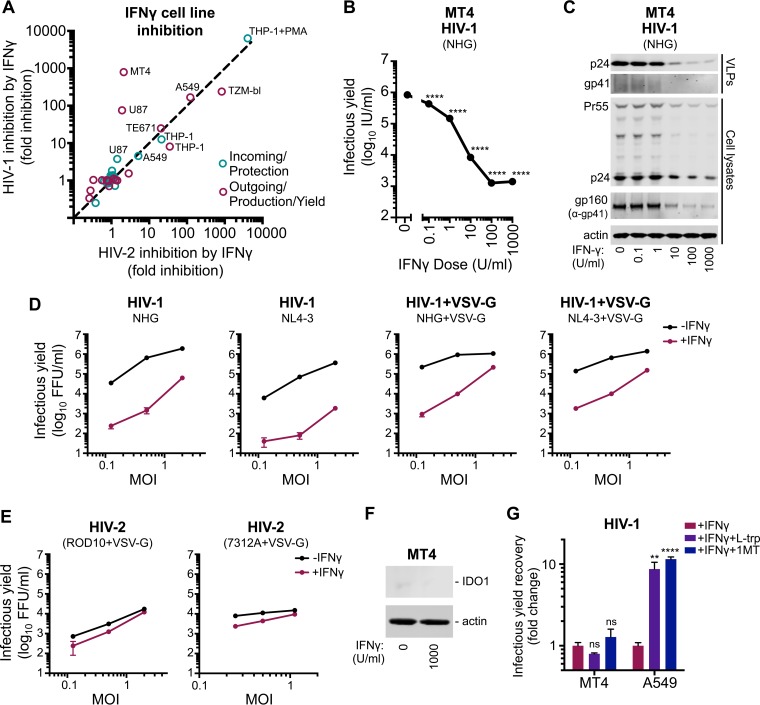
MT4 cells display an HIV-1-specific block that is independent of both route of viral entry and IDO1. (A) Cell line protection from infection (blue circles, using NHGΔenv-GFP and HIV-2_ROD_Δenv-GFP) or the reduction in infectious yield (maroon circles) for HIV-1 and HIV-2 using data from Fig. 2 and 3. (B) The infectious yield of HIV-1 (NHG) from MT4 cells pretreated with various doses (0 to 1000 U/ml) of IFN-γ was quantified as described in the legend of Fig. 3A. (C) Viral antigens present in particulate material or cell lysates were measured by Western blotting using the samples from panel B. (D and E) The infectious yield of IFN-γ-pretreated (1,000 U/ml) or untreated MT4 cells resulting from either HIV-1 (D) or HIV-2 (E) infection at various multiplicities and in the presence or absence of VSV-G pseudotyping was determined through titration of the harvested indicated virus on TZM-bl indicator cells as described in the legend of Fig. 3C. (F) IDO1 expression was monitored in MT4 cells with or without IFN-γ via Western blotting. (G) The fold increase in the yield of infectious HIV-1 from IFN-γ-pretreated MT4 (NHG) or A549 (VSV-G pseudotyped NHG) cells in the presence of 50 μg/ml exogenous l-tryptophan (L-Trp) or 100 μg/ml of the IDO1 competitive inhibitor 1-methyl-l-tryptophan (1MT) was determined as described for panel B. All errors bars indicate SEM. Statistical analyses were performed using unpaired, two-tailed *t* tests (*n* = 3 to 5) (****, *P* < 0.0001; **, *P* < 0.01; ns, not significant, *P* > 0.05).

Remarkably, the stage of the viral life cycle inhibited by IFN-γ was superficially similar in all the cell lines exhibiting late blocks. Viral Gag and capsid (CA) expression levels were reduced in infected cells, and there was a concomitant reduction in the yield of pelletable CA protein in the supernatant (Fig. 3B). Thus, the diminished infectious yield caused by IFN-γ is predominantly manifested as a reduction in viral gene expression and a paucity of progeny virions rather than the retention or inactivation of abundant nascent viral particles. Strikingly, the yield of infectious HIV-1 was markedly reduced in IFN-γ-treated MT4 cells (>500-fold), whereas HIV-2 largely escaped this inhibition (Fig. 3C). The specificity of this block was especially apparent when the magnitudes of HIV-1 and HIV-2 inhibition were compared for all the tested cell lines (Fig. 4A), as the late block to HIV-1 replication in MT4 cells appears as an outlier (although HIV-1-specific inhibition was also apparent in U87 cells to a lesser extent).

### The IFN-γ-induced late block to HIV-1 replication in MT4 cells is independent of IDO1 and viral entry route.

The large magnitude and specific nature of the late block in MT4 cells led us to consider this phenotype in more depth. The late block in MT4 cells was dose dependent, with 10 U/ml of IFN-γ sufficient to block HIV-1 by ∼100-fold and IFN-γ concentrations ≥10 U/ml suppressing both viral protein expression and the genesis of progeny virions (Fig. 4B and C).

Because HIV-1 (NHG) was inhibited in IFN-γ-treated MT4 cells while HIV-2 (VSV-G pseudotyped ROD10) was largely resistant (Fig. 3), we considered whether apparent resistance to late inhibition could be conferred by the envelope or entry pathway used for initial infection. Thus, we examined whether pseudotyped HIV-1 (NHG and NL4-3) was able to replicate in IFN-γ-treated MT4 cells. IFN-γ stimulation potently blocked the replication of HIV-1, using a range of multiplicities of infection, even when the inoculum was pseudotyped with VSV-G (Fig. 4D). Due to the coreceptor tropism of HIV-2 ROD10 and 7312A strains, we could only efficiently infect MT4 cells with pseudotyped HIV-2. However, in contrast to HIV-1, both of the HIV-2 strains we tested were strikingly resistant to IFN-γ-mediated inhibition (Fig. 4E). Thus, using a VSV-G envelope for initial infection does not circumvent the late block in MT4 cells, and at least two HIV-2 strains were largely resistant to this inhibition.

We previously reported that IFN-γ-induced IDO1 expression inhibits HIV-1 replication through tryptophan depletion (38). Although the late block in MT4 cells appears superficially similar to that induced by IDO1, IDO1 expression was not induced in IFN-γ-treated MT4 cells (Fig. 4F), and inhibition of IDO1 (via 1-methyl-l-tryptophan or exogenous l-tryptophan) did not reverse the inhibition of HIV-1 in MT4 cells, unlike in A549 cells (Fig. 4G). Thus, the late IFN-γ-induced block in MT4 cells is entirely independent of IDO1.

### Multiple HIV-1 strains are immune to the IFN-γ-induced late block in MT4 cells.

**FIG 5:**
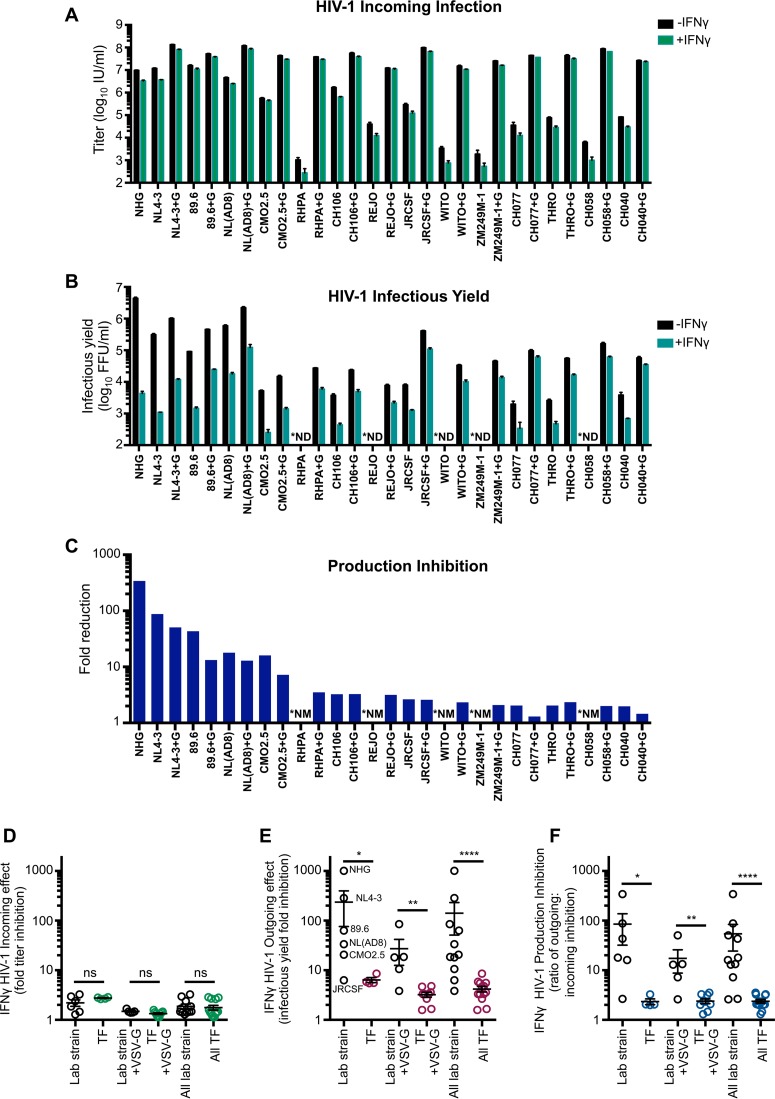
The late IFN-γ-induced block in MT4 cells is HIV-1 strain specific. (A) CCR5^+^ MT4-LTR-GFP (MT4-R5-LTR-GFP) indicator cells were untreated or pretreated (24 h) with 1,000 U/ml of IFN-γ prior to titrated challenge with a panel of HIV-1 infectious molecular clones with and without VSV-G pseudotyping. (B) The infectious yield of HIV-1 (pseudotyped and nonpseudotyped, as indicated) produced from MT4-R5-LTR-GFP cells with or without IFN-γ pretreatment was determined through titration of the indicated progeny virus (harvested at 46 to 48 h postinfection) on TZM-bl indicator cells. (C) To quantify the IFN-γ-mediated block to HIV-1 infectious virus production while taking into account weak early blocks to infection, the mean fold reduction or inhibition in production (normalized fold reduction) was calculated by dividing the mean reduction in infectious yield (fold change determined in panel B) for each virus by the mean protection from infection (fold change determined in panel A) for that virus. (D to F) Representations of the incoming, infectious yield, and production inhibition IFN-γ-mediated effects between HIV-1 lab strain and TF viruses, as indicated, using the data from panels A to C. Designations of TF (versus lab strain) viruses can be found in Table 2. In panels A to C, ^+^G indicates viruses which have been VSV-G pseudotyped, *ND indicates viruses in which we were unable to measure the infectious yield as the incoming infectious titer was too low (without VSV-G pseudotyping), and *NM indicates not measurable (due to not detectable portion of calculation). Error bars indicate SEM (*n* = 3 to 5). Statistical analyses in panels D to F were performed using Mann-Whitney tests (****, *P* < 0.0001; **, *P* < 0.01; *, *P* < 0.05; ns, not significant, *P* > 0.05).

To consider if other HIV-1 strains were also sensitive to this late block in MT4 cells, we examined divergent HIV-1 viruses, including multiple transmitted/founder (TF) clones. Because the majority of HIV-1 viruses, including the TF viruses, infect CD4^+^ CCR5^+^ T cells (44), we used clonal MT4 cells modified to express CCR5. In addition, these cells were further modified to express humanized Renilla reniformis-derived GFP (hrGFP) in response to Tat expression, allowing the levels of HIV-1 infection to be quantified (45). IFN-γ treatment conferred similar, but modest, protection from incoming infection for all HIV-1 viruses in these cells (Fig. 5A and D). In contrast, when HIV-1 production was analyzed, substantial variation in IFN-γ sensitivity was observed in these cells. Multiple group M HIV-1 viruses [NL4-3, NHG, NL(AD8), and 89.6] were potently blocked, and group O HIV-1 (CMO2.5) was also inhibited (to a lesser extent). In contrast, JRCSF and all the TF viruses we tested were largely resistant to IFN-γ-mediated inhibition (Fig. 5B and E). Indeed, for TF viruses, the magnitude of inhibition of infectious HIV-1 production was similar to the relatively small reduction in susceptibility to infection, suggesting that these viruses are almost entirely resistant to the late block in MT4 cells (Fig. 5C and F). We thus conclude that the late block to HIV-1 replication in MT4 cells is highly specific and efficiently evaded/antagonized by TF viruses and might therefore recapitulate a block that TF viruses have been selected to overcome during natural transmission (15, 16).

### Envelope determines transmitted/founder virus resistance to the IFN-γ-induced late block.

**FIG 6:**
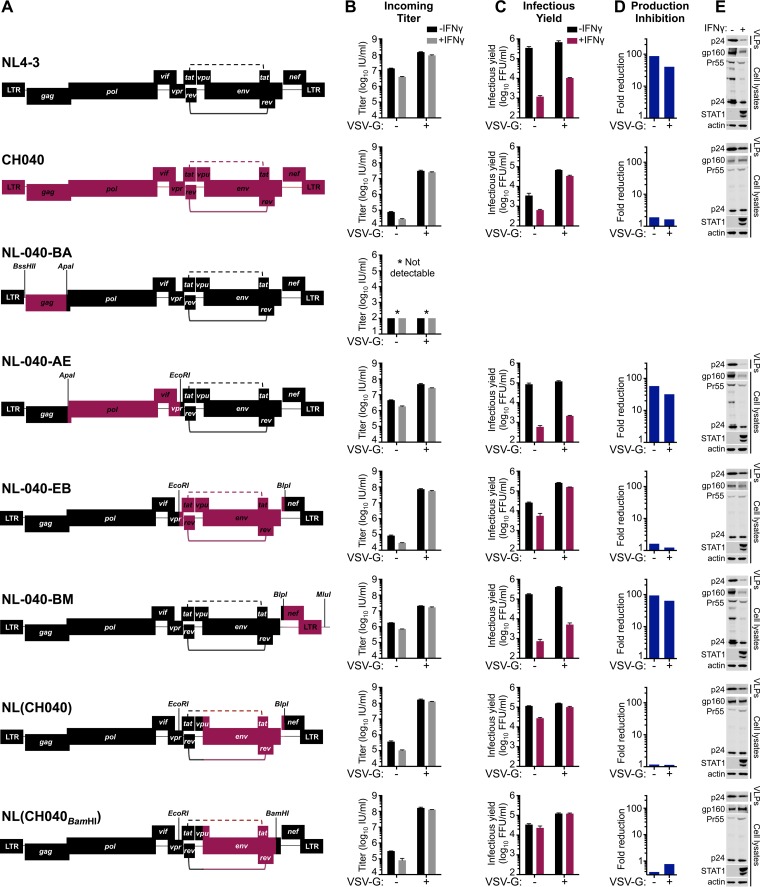
Sensitivity/resistance to the late IFN-γ-induced block in MT4 cells maps to the *env* gene. (A) Representations of the chimeric and parental infectious molecular clones of NL4-3 and CH040 used in the experiments shown in panels B to E. The restriction sites used to clone the chimeras are shown. The incoming titer (B), the yield of infectious progeny (C), the mean fold reduction in infectious HIV-1 production (calculated as described in the legend of Fig. 5) (D), and expression levels of the particulate supernatant capsid and cellular Gag/capsid, gp160, and phosphorylated STAT1 (E) were assessed using MT4-R5-LTR-GFP cells in the presence/absence of IFN-γ pretreatment (as well as VSV-G), as described in the legends of Fig. 4 and 5. The Western blots shown in panel E are those from the VSV-G pseudotyped infections to ensure readily measurable Gag expression. An asterisk (*) indicates that pNL-040-BA was reproducibly severely attenuated, and we were unable to detect infection/replication using this clone. Error bars indicate SEM (*n* = 3 to 5).

In order to map the determinant(s) that confer resistance to IFN-γ-mediated inhibition in MT4 cells, we made a series of chimeric viruses between NL4-3 (sensitive to inhibition) and the TF CH040 (relatively resistant to inhibition). These chimeras were constructed using unique restriction sites in the NL4-3 proviral clone and are represented in Fig. 6A. Unfortunately, one of the clones, chimera NL-040-BA, which contained the majority of *gag* from CH040, was reproducibly severely attenuated, and we were unable to assess the IFN-γ sensitivity of this virus. Notably, IFN-γ resistance mapped to the *env* gene, and chimera NL(CH040_BamHI_), which contains the majority of the TF CH040 envelope (start codon to BamHI site), was almost completely resistant to the IFN-γ-induced late block to HIV-1 replication in MT4 cells and produced abundant nascent infectious particles despite IFN-γ treatment (Fig. 6A to E). Furthermore, analysis of phosphorylated STAT1 in cell lysates indicated that envelope-mediated IFN-γ resistance occurred downstream of STAT1 phosphorylation (Fig. 6E).

**FIG 7:**
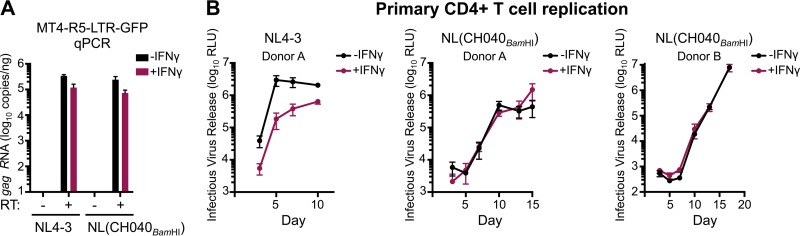
The late IFN-γ-induced inhibition occurs posttranscriptionally and is recapitulated in primary CD4^+^ T cells in a manner rescued by CH040 *env*. (A) The number of copies of transcribed *gag* (following subtraction of cell-associated viral RNA, all measured by qPCR) in the presence or absence of reverse transcriptase (RT) for NL4-3 and NL(CH040_BamHI_), either with or without IFN-γ pretreatment (24 h) in MT4-R5-LTR-GFP cells. (B) Infectious yield of NL4-3 and NL(CH040_BamHI_) from primary CD4^+^ T cells with or without IFN-γ at 3 to 17 days, at an MOI of 0.1 (donor A) or 0.05 (donor B). Yield was determined by TZM-bl assay (*n* = 4 per donor). The IFN-γ sensitivity of donor B to lab strain HIV-1 is shown in Fig. 1G. All error bars indicate SEM.

Subsequent analysis of HIV-1 transcription indicated that IFN-γ treatment reduced HIV-1 transcription by ∼3-fold, a magnitude similar to that of the weak early block observed in MT4 cells (Fig. 6B and 7A). Moreover, NL4-3 and NL(CH040_BamHI_) transcription was similarly influenced by IFN-γ treatment. Thus, the IFN-γ-induced late block likely occurs posttranscriptionally, and CH040 envelope-mediated resistance is not conferred by enhanced transcription. Importantly, CH040 envelope-mediated resistance was also recapitulated in primary CD4^+^ T cells, where NL(CH040_BamHI_) was also more resistant to IFN-γ than NL4-3 (Fig. 7B) or NHG (Fig. 1F and G).

**FIG 8:**
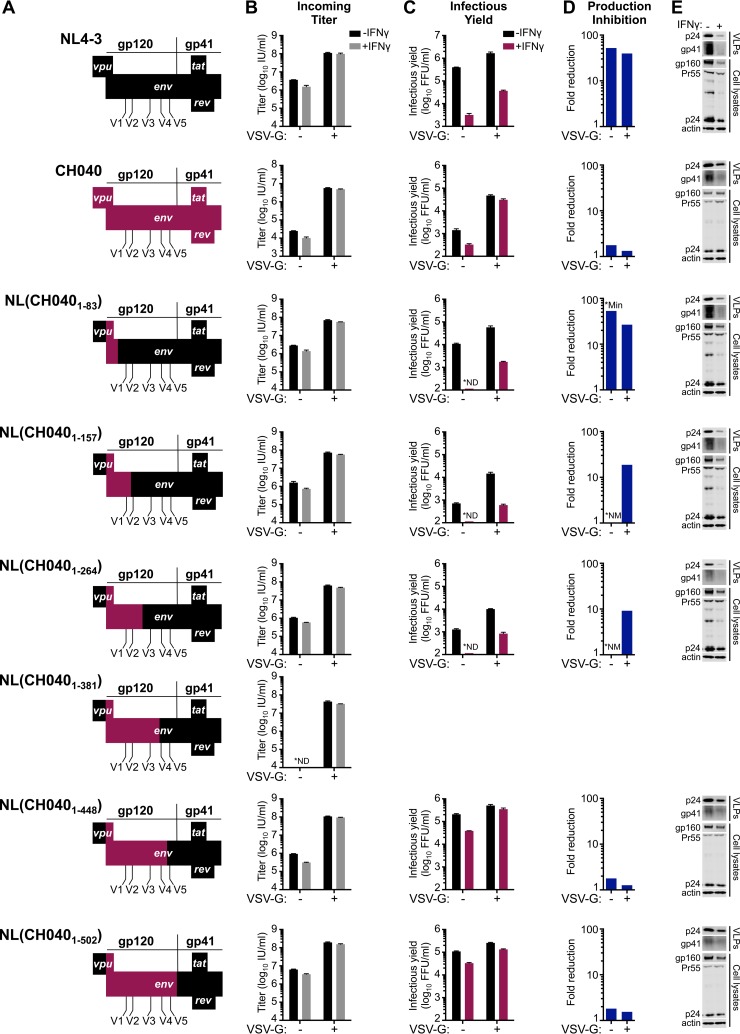
Sensitivity/resistance to the late IFN-γ-induced block in MT4 cells maps to regions of *env* preceding V5. (A) Representations of the *env* chimeric and parental infectious molecular clones of NL4-3 and CH040 used in the experiments shown in panels B to E, with portions of NL4-3 shown in black and portions of CH040 shown in maroon. Portions of the chimeric clones not shown are derived from NL4-3. The incoming titer (B), the yield of infectious progeny (C), the mean fold reduction in infectious HIV-1 production (calculated as described in the legend of Fig. 5) (D), and the particulate supernatant capsid and gp41 and cellular Gag/capsid and gp160 were assessed using MT4-R5-LTR-GFP cells in the presence/absence of IFN-γ pretreatment (as well as VSV-G) (E), as described in the legend of Fig. 6. The Western blots shown in panel E are those from the VSV-G pseudotyped infections to ensure readily measurable Gag expression. pNL(CH040_1–381_) appeared to be reproducibly unable to produce infectious particles, as no infectious particles were detected in the infectious yield assay, and no incoming titer could be detected in the absence of VSV-G. *ND, not detectable; *NM, not measurable (due to not detectable portion of calculation); *Min, minimum possible amount of inhibition due to the undetectable portion of the calculation. All error bars indicate SEM (*n* = 3 to 5).

Finer mapping using additional chimeric viruses in IFN-γ-treated MT4 cells indicated that resistance was largely determined by a region of CH040 gp120 encompassing amino acids 157 to 448 (Fig. 8 and Table 1). Importantly, no Rev-responsive sequences or *tat* or *rev* coding sequences reside in this region of envelope, suggesting that the underlying mechanism of envelope-mediated IFN-γ resistance is likely Tat/Rev independent.

**TABLE 1:** Characterization of IFN-γ-induced changes in nascent particles of sensitive and resistant viruses^*a*^

Virus	Particulate p24 reduction (fold)^*b*^	Particulate gp41 reduction (fold)^*c*^	Relative change in Env per particle^*d*^
NHG^*e*^	20.7	25.3	0.8
NHG T192 M	9.4	6.4	1.5
NL4-3^*e*^	17.0	20.7	0.8
CH040	3.2	2.0	1.6
NL(CH040_1–502_)	2.9	1.4	2.1

a IFN-γ-induced reductions in antigen expression as measured by Li-COR quantitative Western blotting (typical blots are shown in Fig. 4 to 10).

b IFN-γ-induced fold reduction in particulate (VLP) p24 (ratio of VLP without IFN-γ p24 to VLP with IFN-γ p24).

c IFN-γ-induced fold reduction in particulate (VLP) gp41 (ratio of VLP without IFN-γ gp41 to VLP with IFN-γ gp41).

d Ratio of the IFN-γ-induced reduction in particulate p24 (second column; footnote *b*) to the reduction in gp41 (third column; footnote *c*). A value of <1 suggests that there is less gp41 per particle following IFN-γ treatment.

e Shown is mean fold change calculated from multiple experiments.

### A single amino acid substitution in the HIV-1 envelope can confer substantial resistance to the IFN-γ-induced late block.

As a complementary approach, we simultaneously examined whether IFN-γ resistance might be selected through serial passage *in vitro*. After three passages (P3) in IFN-γ-treated MT4 cells, HIV-1 appeared to have adapted and replicated far more efficiently in the presence of IFN-γ, with the IFN-γ-induced delay in HIV-1 overwhelming the culture reduced from >1 week to ∼1 to 2 days (Fig. 9A). Following passage 5 (P5), the proviral DNA from the IFN-γ-treated cells was PCR amplified and sequenced. Visual inspection of the chromatograms (Fig. 9B and C) identified two polymorphisms in *vif* and one polymorphism in *env* that were present in the viral swarm. One synonymous G/A polymorphism present at G5310 and a single nonsynonymous C/T polymorphism present at C5387 encoding either serine or leucine at S116 were present in Vif. L116 does not commonly occur in Vif sequences derived from infected patients, but serine, threonine, and alanine residues are all common at this site (46). Perhaps more importantly, of the three total polymorphisms that were detected in the swarm propagated in the presence of IFN-γ, only one was selected to uniformity (Fig. 9B and C). This single transition, C6795T, encodes a nonsynonymous substitution in variable region 2 (V2) of the envelope glycoprotein (Env), T192M.

**FIG 9:**
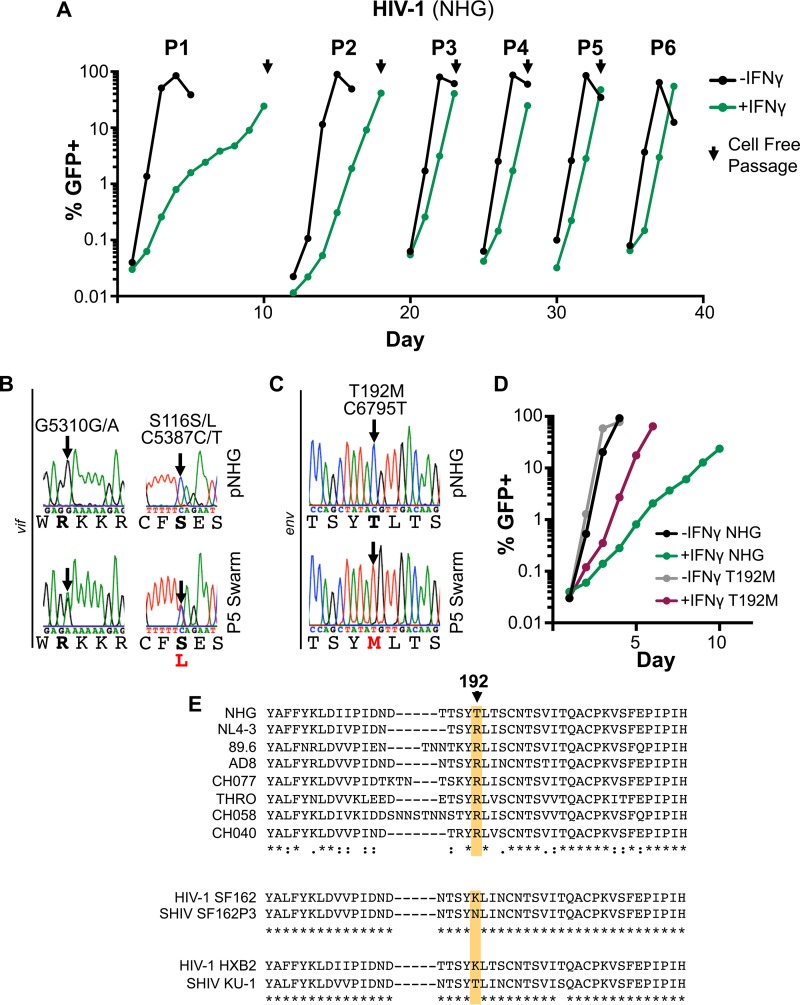
Single substitutions in the HIV-1 envelope can substantially alter susceptibility to the late IFN-γ-induced block in MT4 cells. (A) MT4 cells with and without pretreatment with 1,000 U/ml of IFN-γ were infected at an MOI of ∼0.001 with HIV-1 (NHG). The percentage of GFP-positive cells over time is shown (determined using flow cytometry). HIV-1 propagated in the presence of IFN-γ was used to inoculate fresh MT4 cells (at the times indicated with arrows) with or without IFN-γ pretreatment. (B and C) Sanger sequencing chromatograms from PCR-amplified proviral DNA from the parental clone or the P5 swarm from *vif* or *env* are shown (single nucleotide polymorphisms are highlighted with arrows). (D) Replication of the T192M mutant and the parental clone was measured as described in panel A. (E) An amino acid alignment of the V2 region of gp120 of the four most IFN-γ-sensitive and -resistant strains from the experiment shown in Fig. 5, along with two pathogenic SHIVs (SF162P3 and KU-1) and the corresponding HIV-1 envelope sequence (SF162 and HXB2, respectively). T192 (NHG) is indicated with an arrow and colored shading.

To investigate whether the T192M substitution in Env conferred resistance to the late IFN-γ-induced block in MT4 cells, we generated this substitution in isolation within the parental clone (NHG). HIV-1 T192M had replication kinetics similar to that of the parental virus in MT4 cells (Fig. 9D). Notably, the T192M virus was partially resistant to IFN-γ-mediated inhibition and overran the culture by day 6, when parallel cultures of the parental virus showed less than 5% infection (Fig. 9D). However, position 192 cannot be the only determinant specifying sensitivity/resistance to the late IFN-γ-induced block. In contrast to NHG, analysis of 5,000 patient sequences (Los Alamos HIV database) revealed that most HIV-1 *env* genes encode arginine at this position (∼80%), with isoleucine (∼7.5%), threonine (∼5.9%), and methionine (4.5%) appearing less commonly and valine, lysine, glycine, serine, alanine, and leucine appearing relatively rarely (<1%). Crucially, both sensitive and resistant variants of HIV-1 encode arginine at this position (Fig. 9E), strongly suggesting that other determinant(s) exist within *env*.

**FIG 10:**
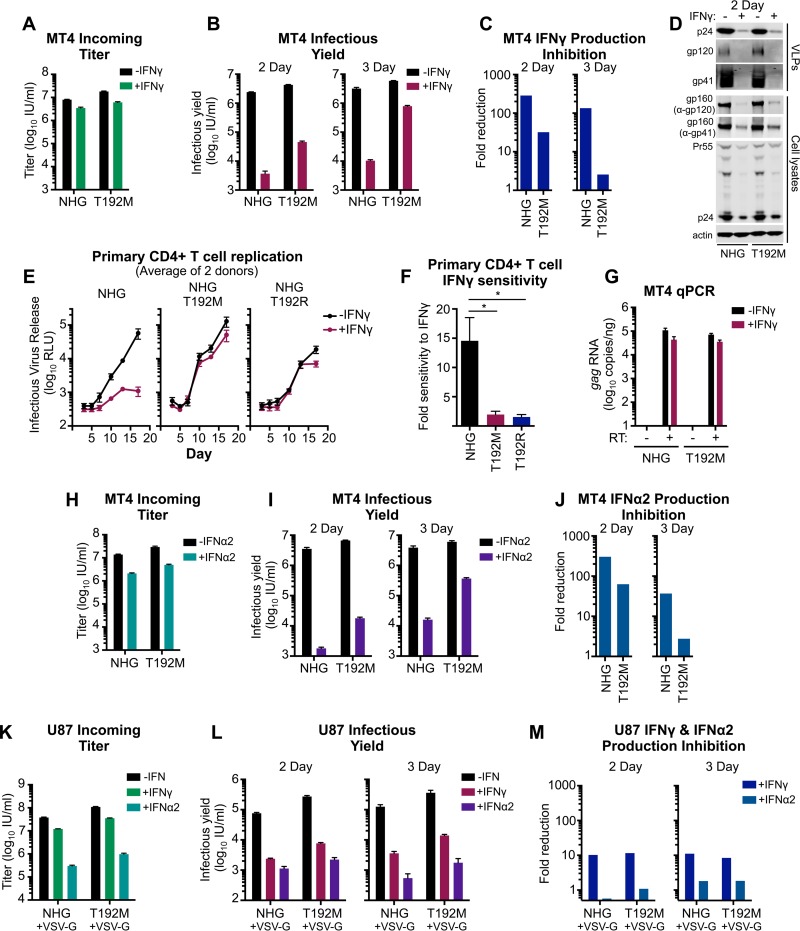
T192M confers partial resistance to late IFN-α2- and IFN-γ-mediated inhibition in MT4 and primary CD4^+^ T cells but not in U87 cells. The infectious titer (A) and infectious yield in the presence and absence of IFN-γ pretreatment (B) as well as production inhibition (C) were assessed for HIV-1 (NHG) and the T192M mutant in MT4 cells, as described in the legend of Fig. 5 (although the virus-containing supernatants used to calculate the infectious yield shown in panel B were titrated on MT4 cells rather than TZM-bl cells, as in the experiment shown in Fig. 3A). (D) Viral capsid and glycoprotein abundance in cell lysates and supernatant particulate matter from the experiment shown in panel B (day 2) was assessed by Western blotting. (E) Infectious yield of NHG, NHG T192M, and NHG T192R from primary CD4^+^ T cells, with or without IFN-γ, at an MOI 0.05 at 3 to 17 days (determined by TZM-bl assay). Shown are average values from two donors (*n* = 4 per donor). (F) Primary CD4^+^ T cell sensitivity to IFN-γ, as determined by ratios of the areas under the curves (AUC) (without IFN-γ AUC/with IFN-γ AUC), using data from panel E. (G) The number of copies of transcribed *gag* (following subtraction of cell-associated viral RNA, all measured by qPCR), in the presence or absence of reverse transcriptase (RT) for NHG and NHG T192M, either with or without IFN-γ pretreatment (24 h), as indicated, in MT4 cells. (H to J) The impact of IFN-α2 pretreatment (24 h) was assessed as in panels A to C in MT4 cells. (K to M) The impact of IFN-γ and IFN-α2 in U87 cells was assessed as in panels A to C but using VSV-G pseudotyped NHG and T192M. All error bars indicate SEM (*n* = 3 to 5). Statistical analyses were performed using unpaired two-tailed *t* tests (*, *P* < 0.05).

We next considered the impact of the T192M substitution on a single round of HIV-1 infection. Importantly, the infectious yield of T192M was >10-fold higher than the yield of the parental virus from IFN-γ-treated MT4 cells (Fig. 10A to D). At 3 days postinfection, the yield of T192M was >50-fold higher than that of the unmodified virus (although this yield arose from more than one round of infection) (Fig. 10B and C). Interestingly, in contrast to the IFN-γ-resistant NL(CH040) chimera (Fig. 6 and 7), the T192M substitution had little impact on the genesis of progeny virions in IFN-γ-treated cells (Fig. 10D and Table 1). The ∼10-fold increase in infectious yield (2 day) was conferred by a marginal <2-fold increase in the abundance of particulate capsid. Thus, T192M appears to increase the infectiousness of nascent virus particles produced from IFN-γ-treated MT4 cells.

Importantly, replication assays using primary CD4^+^ T cells indicated that both T192M and T192R viruses were far more IFN-γ resistant than the parental virus (Fig. 10E and F). Not only does this suggest that the molecular details of the block in MT4 cells are recapitulated in primary cells, but it also highlights the important selection pressure this block may represent *in vivo* and underlines the likely importance of residue 192 during HIV-1 transmission. Furthermore, in accordance with our observations using HIV-1 NL4-3, the late IFN-γ-induced block to HIV-1 NHG replication appeared to occur posttranscriptionally in MT4 cells as IFN-γ treatment inhibited transcription at a magnitude similar to that of the weak early block in these cells. Moreover, the T192M substitution did not enhance transcription in the presence of IFN-γ relative to the parental virus (Fig. 10G).

We next examined whether T192M might also confer resistance to type I IFN treatment in MT4 cells. IFN-α2 and IFN-γ both induced potent late blocks to HIV-1 production in these cells (Fig. 10A to C and H to J). Strikingly, the T192M substitution was able to confer substantial resistance to IFN-α2-induced production inhibition (Fig. 10I and J). Thus, it is possible that type I and type II IFNs inhibit HIV-1 through a common molecular mechanism in MT4 cells. However, it is also possible that increased particle infectiousness confers resistance to multiple blocks that are mechanistically distinct. Importantly, these observations demonstrate that the HIV-1 Env can be a major determinant in governing sensitivity/resistance to both IFN-γ- and IFN-α-induced blocks to HIV-1 replication. Moreover, single amino acid substitutions can substantially alter the sensitivity to IFN-mediated inhibition.

We finally considered whether the T192M substitution might also confer IFN resistance in U87 cells, in which type I IFN is known to inhibit HIV-1 infection (12) and in which HIV-1 is also inhibited with some specificity (Fig. 4A). Interestingly, in U87 cells, IFN-α2 induces an early block to HIV-1, likely mediated by Mx2 (32) and IFITMs (30), whereas IFN-γ induces a late block (Fig. 10K to M). Crucially, the T192M substitution did not confer the same resistance to the IFN-mediated inhibition of HIV-1 production (Fig. 10K to M) that was conferred in MT4 cells and primary CD4^+^ T cells (Fig. 10A to J). Thus, it is likely that the mechanism of IFN-γ-induced late inhibition in U87 cells is mediated by a mechanism that is distinct from the inhibition in primary CD4^+^ T cells and MT4 T cells.

## DISCUSSION

We have examined the often overlooked ability of IFN-γ to inhibit HIV-1 replication and have demonstrated that the HIV-1 *env* gene, including a single amino acid therein, can determine sensitivity/resistance to IFN-γ-mediated inhibition in CD4^+^ T cells. Importantly, TF viruses resisted IFN-γ inhibition, suggesting that IFN-γ-stimulated genes might constrain HIV-1 transmission. In the process, we have also revealed that VSV pseudotyping can seemingly confuse the interpretation of specific inhibition phenotypes.

Unexpectedly, despite efficient Mx2 expression, IFN-α2 did not protect THP-1 cells from HIV-1 infection unless the HIV-1 virus-like particles (VLPs) were decorated with a VSV-G envelope. Thus, we conclude that IFN-α2- or IFN-α14-stimulated Mx2 expression is not sufficient to inhibit HIV-1 (NHG) in THP-1 cells. Crucially, NHG is sensitive to Mx2 inhibition in other contexts (45). These data are consistent with a recent report which suggested that endogenous Mx2 cannot inhibit HIV-1 in type I IFN-treated THP-1 cells (47) but are inconsistent with multiple other studies (32, 33, 48). These previous studies utilized VSV pseudotyped HIV-1, and more work (such as the role of type I interferon subtype or HIV-1 strain) is needed to clarify this apparent discordance.

Although the route of entry determined IFN inhibition in THP-1 cells, in MT4 cells IFN-γ treatment reduced the genesis of infectious progeny virions [as opposed to blocking early stage(s) of the HIV-1 life cycle], regardless of entry route. Nevertheless, the ability to confer sensitivity/resistance to a postintegration block prior to the formation of nascent particles unexpectedly resided in the *env* gene. Although the full mechanistic basis of this IFN-mediated inhibition and resistance is unclear, there has been a recent resurgence of interest in Env as a target for antiviral host genes, with both GBP5 and MARCH8 proposed to inhibit HIV-1 by interfering with the correct processing and incorporation of Env glycoproteins (31, 49). Moreover, the TF envelope defines resistance to an early block caused by IFITM proteins (30). Crucially, GBP5 and MARCH8 reduce the infectivity of HIV-1 particles as opposed to preventing the genesis of nascent particles and thus cannot explain the late block observed here. In addition, IFITM proteins present an early block (30) or reduce the infectivity of nascent particles (28, 29) and also cannot explain the late block we have observed here (which manifests as reduced viral protein expression and attenuated virion biogenesis).

More work is required to ascertain the relative contribution that particle infectiousness and efficient viral gene expression might make in conferring the strain-specific IFN resistance we observe here. Nevertheless, certain patterns regarding particulate antigen expression are evident. For example, it is clear that for IFN-γ-sensitive viruses (like NHG and NL4-3), at least 90 to 95% of the large reduction in infectious yield arises from the reduction in nascent particle production (i.e., the 17- to 20-fold reduction in VLP p24) (Table 1) rather than from a loss of infectiousness. Likewise, the more IFN-γ-resistant viruses that include the CH040 envelope, or even just a portion of CH040 gp120, are better able to sustain particle production in the face of IFN-γ treatment (i.e., only ∼3-fold reduction in VLP p24) (Table 1). Still, IFN-γ-resistant particle production and increased particle infectiousness are not mutually exclusive, and we note that all of the IFN-resistant viruses we analyzed exhibited relatively minor decreases in virion-associated gp41 compared to IFN-sensitive viruses (Table 1). In particular, increased envelope incorporation may explain how the T192M substitution confers IFN-γ resistance by increasing the infectiousness of individual particles (∼10-fold gain in infectivity is conferred by a modest 1- to 2-fold increase in pelletable p24). It is also possible that viruses containing the CH040 envelope retain higher particle infectivity in the face of IFN-γ treatment (compared to IFN-sensitive viruses). In this regard, TF viruses are known to more efficiently incorporate viral glycoproteins and have higher particle infectivity than chronic control viruses, highlighting that infectiousness could impact IFN resistance (15). Yet the general lack of linearity between infectivity data and single-dose Western blot quantification urges caution in the interpretation of smaller differences in antigen expression, and although we do not rule out the role of particle infectivity or fusogenicity in CH040 envelope-mediated resistance, the role of particle production does appear more immediately evident.

Notably, the HIV-1 strains that are sensitive to the late block in MT4 cells are derived from passaged lab stocks, whereas the resistant viruses are, with one exception, TF viruses. The exception is JRCSF, an isolate from the cerebrospinal fluid of an AIDS patient, which was minimally passaged for 11 days in peripheral blood mononuclear cells (PBMCs) prior to molecular cloning (50). Interestingly, JRCSF has been previously reported to have a replication profile similar to that of TF viruses (51). Yet with the relatively small sample of HIV-1 strains tested here, we are unable to conclude that only TF viruses resist the late IFN-induced block in MT4 cells. Whether IFN sensitivity is a property gained through *in vitro* passage or whether sensitivity arises in certain chronic virus lineages remains to be determined.

Likewise, our study has also not yet ruled out that the anti-HIV-1 activity of IFN-γ could be indirect (mediated by an IFN-γ-stimulated cytokine). Moreover, whether the IFN-γ-induced inhibition observed in primary cells is phenotypically analogous to the block in MT4 cells (occurring late in the viral life cycle) has also not yet been determined. However, regardless of the details of the signaling events or inhibitory mechanism downstream of IFN-γ stimulation, the fact that the envelope of transmitted HIV-1 can confer remarkable IFN resistance in primary cells suggests that these observations may be of importance *in vivo*.

Interestingly, the same HIV-1 Env residue that was selected during passage in the presence of IFN-γ and conferred IFN-γ resistance in primary cells, 192, was recently identified as a signature site that significantly differed in early versus chronic viruses. While early viruses were overwhelmingly arginine (R192), this residue was significantly less represented during chronic infection (52, 53). Clearly residue 192 is not the only position governing sensitivity/resistance to the late IFN-induced block in MT4 cells as both sensitive and resistant viruses encode arginine at this position, and additional resistance determinant(s) likely reside within *env*. However, given that the site identified during *in vitro* adaptation is apparently enriched during transmission or early infection, it appears that the factor(s) that mediate the late block described here may influence HIV-1 transmission and/or pathogenesis. Moreover, the observation that all of the TF viruses largely resisted late IFN-γ-mediated inhibition in MT4 cells, while other strains were robustly (>100-fold) inhibited, suggests that the molecular basis of this block may also be recapitulated *in vivo*.

Strikingly, substitutions at position 192 that conferred resistance to inhibition in both MT4 and primary CD4^+^ T cells were also selected during the distinct *in vivo* passaging of simian-human immunodeficiency viruses (SHIVs) SF162P3 and KU-1 (54, 55) (Fig. 9E). These are the only pathogenic SHIVs we are aware of whose parental *env* gene did not encode arginine at this position, and both adapted during passage *in vivo*. Importantly, this *in vivo* adaptation took place by infecting macaques for 2 to 16 weeks, a time frame when IFN responses and IFN-induced selection are likely most active (1). In accordance with this idea, Boyd et al. recently reported that adapting pathogenic SHIVs *in vivo* also selects for *env*-mediated IFN-resistance (56).

We have demonstrated that IFN-γ can robustly inhibit HIV-1 in both primary cells and cell lines and have shown that the HIV-1 *env* gene appears to play a key role in governing the sensitivity/resistance of HIV-1 to IFN-mediated inhibition. The factor(s) that mediate this block and how transmitted HIV-1 escapes inhibition are currently unknown. Understanding these events could shed light on the critical and vulnerable bottleneck that occurs during HIV-1 transmission.

## MATERIALS AND METHODS

### Cell lines.

We assembled a panel of cell lines that are commonly used in HIV-1 research and/or were readily available from the UK National Institute of Biological Standards and Control (NIBSC). Jurkat, THP-1, HEK 293T, TE671 (RD or TE671/RD), and TZM-bl cell lines were a generous gift from Paul Bieniasz. MONOMAC6 cells were a generous gift from Mark Marsh, and A549 cells were a generous gift from Ben Hale. The following cell lines were obtained from the UK NIBSC (repository reference indicated in parentheses): Molt 4 clone 8 (ARP052), H9 (ARP001), HUT78 (ARP002), MOLT3 (ARP010), MOLT4 (ARP011), U937 (ARP012), HL60 (ARP030), PM1 (ARP057), AA-2 (ARP054), KARPAS 45 (ARP032), and U87 (U87.MG, ARP 043). Human suspension cell lines were maintained in RPMI medium supplemented with 9% fetal calf serum (FCS) and gentamicin, with the exception of MONOMAC6 cells whose culture medium was also supplemented with nonessential amino acids (NEAA) and OPI medium supplement (Sigma-Aldrich). The remaining cell lines were cultured in Dulbecco's modified Eagle's medium (DMEM) supplemented with 9% FCS and gentamicin. All cell lines were tested for mycoplasma contamination, and the KARPAS 45 cell line tested positive for mycoplasma at the end of this study and should be considered mycoplasma positive throughout this study. The CCR5^+^ MT4-R5-LTR-GFP (where LTR is long terminal repeat) indicator cells were described previously and contain a cassette in which hrGFP expression is driven by the HIV-1 LTR (38, 45).

PMA-treated THP-1 cells were seeded with 30 ng/ml of PMA in RPMI medium plus 9% FCS and gentamicin overnight and washed the following morning (∼16 h later) to remove the PMA.

### Primary cells.

As described previously (30), human primary CD4^+^ T cells were obtained from peripheral blood mononuclear cells (PBMCs) from healthy human donors. Ethical approval for primary cell work was granted by King's College London Infectious Disease BioBank Local Research Ethics Committee (under the authority of the Southampton and South West Hampshire Research Ethics Committee, approval REC09/H0504/39), approval number SN-1/6/7/9. In brief, PBMCs were isolated by density gradient centrifugation through Lymphoprep (Axis-Shield), and CD4^+^ T cells were obtained by negative selection using a Dynabeads Untouched Human CD4 T Cells kit (Life Technologies) according to the manufacturer's instructions. Flow cytometry for CD4 was used to assess the purity of the isolated cell population, which was reproducibly >95%. Cells were cultured with RPMI medium supplemented with 10% FCS, 20 μg/ml gentamicin, and 30 U/ml recombinant interleukin-2 (IL-2) (PeproTech) and were then activated within 48 h using Dynabeads Human T-Activator CD3/CD28 beads (CD3/CD28 Dynabeads; Invitrogen) according to the manufacturer's instructions. Cells were typically resuspended in flasks with the CD3/CD28 Dynabeads at a 1:5 bead-cell ratio (cells at 1 × 10^6^ cells/ml). Cells were washed and resuspended in fresh RPMI medium supplemented with IL-2 prior to infection or postactivation analysis. Where indicated on specific figures or in the figure legends or text, cells were treated with IFN-γ (1,000 U/ml human interferon gamma) (PHC4031; Thermo Fisher) for 24 h before virus infection.

### Primate lentiviruses.

Lentivirus stocks (Table 2) were all generated through transient transfection of HEK 293T cells in the presence/absence of pCMV-VSV-G using polyethylenimine (PEI). The following proviral clones were used: pNL4-3 (GenBank accession number M19921.2) (57), pROD10 (KY272752) (58), pJK7312A (L36874) (59), pCMO2.5 (AY623602.1) (60), and replication-competent GFP-encoding pNHG (JQ585717) (61, 62). The following HIV-1 strains were obtained from the NIH AIDS Reagents Program (catalog numbers indicated in parentheses): pNL(AD8) (11346), pYK-JRCSF (JRCSF, 2708), p89.6 (3552), pZM249M-1 (12260), and a panel of full-length transmitted/founder (TF) HIV-1 infectious molecular clones (11919). In addition to chimeric/mutant proviral clones, *env*-defective GFP-encoding NHGΔenv-GFP (61, 63) and pHIV-2_ROD_Δenv-GFP (33) were also used. In all cases, supernatants were harvested at ∼48 h posttransfection and clarified using a 0.45-μm-pore-size filter.

**TABLE 2:** Viruses used in this study

Virus (subtype) and designation	Description or catalog no.^*a*^	GenBank accession no(s).	TF	Coreceptor tropism	Reference(s)
HIV-1 Group M (B)					
NHG	GFP in place of *nef*	JQ585717	No	X4	61, 62
NHGΔenv-GFP	Lacks a functional *env* gene, GFP in place of *nef*	NA^*c*^	No	—^*d*^	61, 63
NL4-3	114	M19921.2	No	X4	57
89.6	3552	U39362	No	R5/X4	69
NL(AD8)	11346	NA	No	R5	70
JRCSF	2708	M38429	No	R5	50
RHPA	11744	JN944944	Yes	R5	51
CH106	11743	JN944942	Yes	R5	51
REJO	11746	JN944943	Yes	R5	51
WITO	11739	JN944948	Yes	R5	51
CH077	11742	JN944941	Yes	R5/X4	51
THRO	11745	JN944946	Yes	R5	51
CH058	11856	JN944940	Yes	R5	51
CH040	11740	JN944939	Yes	R5	51
HIV-1 Group M (C)					
ZM249 M-1	12260^*b*^		Yes	R5	15, 71
HIV-1 Group O					
CMO2.5		AY623602.1	No		60
HIV-2					
HIV-2_ROD_Δenv-GFP	Lacks a functional *env* gene, GFP in place of *nef*	NA	No	—^*d*^	33
ROD10		M15390, KY272752	No		58
7312A		L36874	No	R5	72

a NIH AIDS Reagent Program catalogue number.

b Sequence available from the NIH AIDS Reagent Program.

c NA, not available.

d Not applicable.

### VSV.

Vesicular stomatitis virus (VSV)-GFP (Indiana serotype) competent to undergo a single round of infection but not encoding the VSV-G envelope (rVSV-ΔG-GFP decorated with VSV-G expressed in *trans*) was used (64). Virus stocks were generated through transfection of HEK 293T cells using PEI (PolySciences) with 2 μg of VSV-G plasmid. The following day, the cells were infected with rVSV-ΔG-GFP at an MOI of 1. Progeny VLP stocks were harvested at 24 h postinfection and clarified using a 0.45-μm filter.

### Plasmid construction of chimeric/mutant viruses.

Chimeric proviral molecular clones between pNL4-3 (GenBank accession number M19921.2) and pCH040 (NIH AIDS Reagent Program 11919) were constructed first by introducing an MluI restriction site outside the proviral sequence in pNL4-3 (in the 3′ host genomic sequence) by inserting the complementary oligonucleotides 5′-CAT GTA CGC GTA AGC TTA-3′ and 5′-CAT GTA AGC TTA CGC GTA-3′ inside the unique NcoI site. The resulting pNL4-3ΔNcoI+MluI was used as a backbone to receive BssHII-ApaI (pNL-040-BA), ApaI-EcoRI (pNL-040-AE), EcoRI-BlpI (pNL-040-EB), and BlpI-MluI (pNL-040-BM) fragments from pCH040.

To generate pNL(CH040) and pNL(CH040_BamHI_), overlap extension PCR was used to weave together the amplified sequence of pNL4-3 between the unique EcoRI site and the start codon of *env* (5′-GAT ACT TGG GCA GGA GTG GAA GCC ATA ATA AGA ATT CTG C-3′ and 5′-CCT GAT CCC CAT CAC TCT CAT TGC CAC TGT CTT CTG CTC TTT CTA TTA G-3′) with the PCR-amplified region of pCH040 between the *env* start codon and the BlpI site (5′-GAG CAG AAG ACA GTG GCA ATG AGA GTG ATG GGG ATC AGG AAG AAT TAT C-3′ and 5′-TAT TGC TAC TTG TGA TTG CTC CAT G-3′) or the amplified region of pCH040 between the start codon and the BamHI site in the NL4-3 *env* (5′-GAG CAG AAG ACA GTG GCA ATG AGA GTG ATG GGG ATC AGG AAG AAT TAT C-3′ and 5′-GAG AGA *GGA TCC* GTT CAC TAA TGG ATC GGA TCT G-3′). CH040 has no BamHI site in *env*, and this site was specified in the oligonucleotide sequence (italicized). PCRs were completed using Pfu Turbo (Agilent) and inserted into pNL4-3ΔNcoI+MluI using EcoRI and BlpI for pNL(CH040) and EcoRI and BamHI for pNL(CH040_BamHI_).

To generate the T192M mutant virus, overlap extension PCR was used to weave a region of pNHG (GenBank accession number JQ585717) amplified using oligonucleotides 5′-GAT ACT TGG GCA GGA GTG GAA GCC ATA ATA AGA ATT CTG C-3′ and 5′-GTA ATG ACT GAG GTG TTA CAA CTT GTC AAC *A*TA TAG CTG GTA GTA TCA TTA TCT ATT GG-3′ together with a region of pNHG amplified using oligonucleotides 5′-CCA ATA GAT AAT GAT ACT ACC AGC TAT A*T*G TTG ACA AGT TGT AAC ACC TCA GTC ATT AC-3′ and 5′-AGA GAG GCG GCC GCT TAT AGC AAA ATC CTT TCC AAG CCC-3′ (italicized nucleotides specify the C6795T/T192M mutation). To generate the T192R mutant virus, overlap extension PCR was also used to weave a region of pNHG (JQ585717) amplified using oligonucleotides 5′-GAT ACT TGG GCA GGA GTG GAA GCC ATA ATA AGA ATT CTG C-3′ and 5′-GTA ATG ACT GAG GTG TTA CAA CTT GTC AAC *C*TA TAG CTG GTA GTA TCA TTA TCT ATT GG-3′ together with a region of pNHG amplified using oligonucleotides 5′-CCA ATA GAT AAT GAT ACT ACC AGC TAT A*G*G TTG ACA AGT TGT AAC ACC TCA GTC ATT AC-3′ and 5′-AGA GAG GCG GCC GCT TAT AGC AAA ATC CTT TCC AAG CCC-3′ (italicized nucleotides specify the C6795G/T192R mutation). The PCRs were completed using Pfu Turbo (Agilent) and inserted into pNHG using EcoRI and BamHI.

To generate CH040 and NL4-3 *env* chimeras, overlap extension PCR was used to weave together a fragment of NL(CH040) amplified using 5′-GAT ACT TGG GCA GGA GTG GAA GCC ATA ATA AGA ATT CTG C-3′ paired with either 5′-TTG GGG TCT GTG GGT ACA CAG GCG TGT GTG GCC CAA ACA TTA TGT G-3′ [NL(CH040_1–83_)], 5′-TTG TGC TGA TAT TGA AAG AGC AGT TTT TTA CTT CTC CCT TCT CCA TC-3′ [NL(CH040_1–157_)], 5′-TGA GTT GAT ACT ACT GGC CTA ATT CCA TGT GTA CAT TGT ACT GTG-3′ [NL(CH040_1–264_)], 5′-GTT GAA TTA CAG TAG AAA AAT TCC CCT CCG CAA TTG AAA CTG TAC-3′ [NL(CH040_1–381_)], 5′-CTC TTG TTA ATA GCA GCC CAG TAA TGT TTG ATG AGC ATC TAA TTT TTC C-3′ [NL(CH040_1–448_)], or 5′-ATT CCC ACT GCT CTT TTT TCC CTC TGC ACC ACT CTC CTC TTT GCC-3′ [NL(CH040_1–502_)] with a fragment of pNL4-3 amplified using 5′-AGA GAG GCG GCC GCT TAT AGC AAA ATC CTT TCC AAG CCC−3′ paired with either 5′-ATG TTT GGG CCA CAC ACG CCT GTG TAC CCA CAG ACC CCA ACC CAC-3′ [NL(CH040_1–83_)], 5′-AAG GGA GAA GTA AAA AAC TGC TCT TTC AAT ATC AGC ACA AGC ATA AG-3′ [NL(CH040_1–157_)], 5′-TAC AAT GTA CAC ATG GAA TTA GGC CAG TAG TAT CAA CTC AAC TGC-3′ [NL(CH040_1–264_)], 5′-GTT TCA ATT GCG GAG GGG AAT TTT TCT ACT GTA ATT CAA CAC AAC-3′ [NL(CH040_1–381_)], 5′-AGA TGC TCA TCA AAC ATT ACT GGG CTG CTA TTA ACA AGA GAT GGT GG-3′ [NL(CH040_1–448_)], or 5′-AGA GGA GAG TGG TGC AGA GGG AAA AAA GAG CAG TGG GAA TAG GAG−3′ [NL(CH040_1–502_)]. PCRs were completed using Pfu Turbo (Agilent) and inserted into pNL4-3ΔNcoI+MluI using EcoRI and BamHI.

### Western blot analyses.

Cell lysates or VLPs were resolved using 4 to 12% acrylamide gels, transferred onto nitrocellulose membranes, and probed with either anti-actin (JLA20 hybridoma, provided by the Developmental Studies Hybridoma Bank at the University of Iowa), anti-Mx2 (SC-47197; Santa Cruz Biotechnology, Inc.), anti-IDO1 (ab55305; Abcam), or anti-phospho-STAT1 (Tyr701) (9171; Cell Signaling Technology) or with anti-CA (183-H12-5C hybridoma), anti-gp41 (Chessie 8 hybridoma), or anti-gp120 (Chessie 13-39.1 hybridoma) from the NIH AIDS Reagent Program (catalog numbers 1513, 526, and 990, respectively). Membranes were then probed with fluorescently labeled goat/donkey secondary antibodies (Thermo Scientific) prior to scanning with a Li-COR Odyssey scanner. Band intensities were quantified using Image Studio software (Li-COR).

### Cell line authentication.

The identities of THP-1, MT4, A549, TE671, and U87 cells (which all exhibited inhibitory IFN-γ phenotypes) were confirmed using short tandem repeat (STR) analysis carried out by the DNA Diagnostics Centre (DDC), United Kingdom, and analyzed using the DSMZ online STR analysis tool. Although we have used the term TE671 throughout the manuscript, the ATCC STR analysis was unable to substantially distinguish between TE671 (ATCC HTB-139) and RD (ATCC CCL-136). Our TE671 cells were identified as the rhabdomyosarcoma cell line RD by the DDC/DSMZ. The TE671 cell line is likely derived from RD cells, and ATCC HTB-139 has been discontinued for this reason (65). We opted to use the term TE671 as it is common in the field; however, TE671/RD or RD more accurately reflects the lineage of this line. The identity of TZM-bl cells was validated using a TZM assay.

### Virus titrations.

Virus titrations were carried out as described previously (38, 45, 62, 66, 67). Briefly, target cells were infected with a titrated challenge of serially diluted virus-containing supernatant. Suspension cell lines were treated with polyanionic dextran sulfate ∼18 h postinfection to limit infection to a single cycle. At 48 h postinfection, the level of infection was determined using flow cytometry (for either GFP-encoding viruses or MT4-R5-LTR-GFP-infected cells), or TZM-bl infected foci were enumerated using an AID ViruSpot reader. In all cases, the value plotted is the mean of at least triplicate (*n* = 3 to 5) estimations of the titer extrapolated from different doses within the linear range (error bars represent the standard errors of the means [SEM]). A typical result from at least two independent experiments is shown.

### Infectious yield assays.

The infectious-yield assays were carried out essentially as described previously (38). Briefly, cells were seeded in six-well plates and treated, as indicated, with IFN-γ (PHC4031; Life Technologies) or pegylated IFN-α2 (ViraferonPeg; Schering-Plough) in the absence or presence of additional l-tryptophan (50 μg/ml) or 1-methyl-l-tryptophan (100 μg/ml), as indicated on the figures or in the figure legends or text. Adherent cell lines were seeded an additional day prior to IFN treatment. The unit dose of IFN-γ was determined using the most conservative estimate given by the manufacturer (2 × 10^6^ units/mg). At 24 h after IFN-γ or IFN-α2 treatment, cells were infected with the virus indicated on the figures or in the figure legends or text at an MOI of 0.5 or at an MOI < 0.5 for viruses in which an MOI of 0.5 was unachievable due to low infectious titers (for non-VSV-G pseudotyped JRCSF, THRO, CH040, and CH077, in which case 1,800 μl of virus-containing supernatant was used) for 6 h, after which the inoculum was removed by washing the cells in PBS. At 46 to 48 h postinfection, cells were lysed in SDS sample buffer, and the virus-containing supernatant was harvested, filtered, and titrated onto TZM-bl cells or MT4 cells as described above, or pelletable material was isolated for Western blotting by centrifugation through a 20% sucrose cushion.

The 46- to 48-h time point for harvesting was based upon a recent analysis of the replication kinetics of HIV-1 in MT4 cells (41). MT4 cells are the most susceptible and permissive target for HIV-1 NHG replication we are aware of (Fig. 2A). Holmes et al. (41) estimated that a typical MT4 cell produces virus 40 to 46 h after infection and that progeny virions are barely detectable at 24 h. Thus, 46 to 48 h is a time point where abundant progeny virions have been produced from the first round of infection but where progeny virions from the second round of infection have not yet had time to accumulate (41). Harvesting at earlier time points (such as 40 h) that could effectively exclude progeny resulting from the second round of infection greatly reduced the infectious titer (41).

### Primary cell assays.

Where indicated on the figures or in the figure legends or text, cells were treated with 1,000 U/ml IFN-γ (PHC4031; Thermo Fisher) for 24 h before virus infection. In all cases, 2 × 10^5^ activated CD4^+^ T cells were infected at an MOI of 0.05 to 0.1 as specified on the figures or in the figure legends or text (infectious titer determined via TZM-bl assay). At 8 to 12 h postinfection, medium was replaced. Supernatants were harvested when indicated on the figures or in the figure legends or text for up to 17 days postinfection, and infectious viral release at each time point was determined by infecting HeLa-TZM-bl indicator cells. At 48 h post TZM-bl infection, virus release was assayed by measuring chemiluminescent β-galactosidase activity using a Tropix Galacto-Star system (Applied Biosystems) according to the manufacturer's instructions.

### HIV-1 *in vitro* evolution, DNA extraction, PCR, and sequencing.

MT4 cells pretreated (24 h with 1,000 U/ml) with IFN-γ or untreated were infected with HIV-1 (NHG) (GenBank accession number JQ585717), and the level of infection was monitored every 24 h using flow cytometry as described previously (45, 62). At the time points indicated on the figures, the IFN-γ-treated virus-containing supernatant was clarified using a 0.45-μm filter and used to infect fresh cultures pretreated with IFN-γ or left untreated. Following passage 5, genomic DNA, laden with proviral insertions, was extracted using a DNeasy kit (Qiagen), and six segments of NHG proviral DNA encompassing all coding regions were PCR amplified using GoTaq polymerase (Promega) and the following primer pairs: 5′-GCG CCC GAA CAG GGA CTT GAA AGC G-3′ and 5′-GGT GGG GCT GTT GGC TCT GGT CTG C-3′, 5′-TGA AAG ATT GTA CTG AGA GAC AGG C-3′ and 5′-TTT CAC ATC ATT AGT GTG GGC ACC C-3′, 5′-CAG AAG CAG GGG CAA GGC CAA TGG AC-3′ and 5′-ACT TGC CAC ACA ATC ATC ACC TGC C-3′, 5′-AAA GCT CCT CTG GAA AGG TGA AGG G-3′ and 5′-ATT TAC CAA TAC TAC TTC TTG TGG G-3′, 5′-GAT GCT AAA GCA TAT GAT ACA GAG G-3′ and 5′-CAG ATG CTG TTG CGC CTC AAT AGC C-3′, and 5′-CGT CAA TGA CGC TGA CGG TAC AGG C-3′ and 5′-TAA GAT CTA CAG CTC ATG AGT TGG C-3′. The amplicons were subsequently sequenced directly using Sanger sequencing (Eurofins Genomics).

### Analysis of HIV-1 transcription by quantitative reverse transcription-PCR (qRT-PCR).

MT4 or MT4-R5-LTR-GFP cells pretreated (24 h with 1,000 U/ml) with IFN-γ or untreated were infected with HIV-1. Cells were harvested using TRIzol reagent (Invitrogen) at 24 h postinfection, and RNA was isolated through a hybrid TRIzol and RNeasy (Qiagen) protocol (including an on-column DNase treatment). PCR template cDNA was generated using Superscript III (Invitrogen) primed using random hexamers in accordance with the manufacturer's instructions. The *gag* RNA copy number was determined using primers and probes described previously (68) that amplify a conserved region of HIV-1 *gag* compared with standards of serially diluted pNHG plasmid DNA. To specifically measure transcribed RNA, for each condition, duplicate samples (where infection was blocked using dextran sulfate) were also analyzed (to enumerate the “input” cell-associated viral RNA). To quantify transcribed RNA, the input viral RNA (vRNA) was subtracted from the total number of *gag* RNA copies in infected cells. Values represent the means of duplicate quantitative PCR (qPCR) determinations of at least three replicate infections (error bars indicate SEM; *n* = 3).

### Analysis of *env* variants.

To determine the frequency of each amino acid at position 192 in HIV-1 *env*, the Los Alamos National Database (http://www.hiv.lanl.gov/) was used to download all *env* gene sequences available. Only one sequence was selected per patient, and 5,000 sequences were selected at random to determine the frequency of different amino acids at envelope position 192.
